# 13.3 EFFECTS OF CANNABINOIDS ON A HUMAN OLIGODENDROCYTE CULTURE: IMPLICATIONS FOR SCHIZOPHRENIA

**DOI:** 10.1093/schbul/sby014.051

**Published:** 2018-04-01

**Authors:** Valéria Almeida, Daniel Martins-De-Souza

**Affiliations:** University of Campinas (UNICAMP)

## Abstract

**Background:**

Preclinical studies have suggested the involvement of the endocannabinoid system in schizophrenia pathobiology. The effects of cannabinoid drugs in several animal models for schizophrenia have been used to understand the pathobiology of the disease, and to investigate potential treatments for schizophrenia symptoms. Alterations in endocannabinoid (eCB) signaling, such as cannabinoid receptor expression and anandamide levels, have also been investigated in animal models. In addition, in vitro studies have shown the molecular pathways and biological processes associated with cannabinoids’ effects in some cell types, such as glial cell cultures. Glial cells, which express cannabinoid CB1 and CB2 receptors and synthesize eCBs, have been shown to be implicated in schizophrenia. Thus, the effects of cannabinoid drugs on these cells may contribute to our knowledge about the pathobiology of schizophrenia. Specifically, oligodendrocytes are associated with white matter deficits in schizophrenia. The modulation of their function, survival, and differentiation can result in new approaches to treat schizophrenia’s white matter-associated deficits. Here we have investigated the effects of cannabidiol (CBD) on a human oligodendrocyte culture (MO3.13) in terms of protein expression.

**Methods:**

MO3.13 oligodendrocytes were treated with CBD (1µM) for 8h. Proteins were extracted from these cells, digested, and processed in a state-of-the-art LC-MS/MS system. Quantitative proteomics approaches were then employed in a label-free fashion. Differentially expressed proteins among the CBD treatment and controls were analyzed using systems biology in silico tools.

**Results:**

Analyses identified that several proteins were up- or down-regulated in response to CBD treatment. These proteins were analyzed in terms of biological processes, pathways, and functions. CBD affected the expression of 136 proteins. Some proteins such as the transient receptor potential channel (TRPM7), microtubule-associated proteins (MAP2 and MAP4), Rho GTPase activating proteins (21 and 23), and calcium channel voltage-dependent T type alpha 1H (CACNA1H), among others possibly involved in schizophrenia pathobiology, were increased by CBD-treatment.

**Discussion:**

Studies have shown the effects of CBD on the treatment of schizophrenia; but the mechanisms involved in its antipsychotic properties are not fully understood. Herein, we observed that CBD modulated the expression of proteins that can be implicated in schizophrenia pathobiology. For instance, MAPs functions are related to cytoskeleton organization, differentiation, and migration of oligodendrocytes. Studies have shown a decrease of MAPs in schizophrenia patients; thus, increasing MAP2 and MAP4 by CBD may be an interesting mechanism to treat and prevent cytoskeleton impairments in oligodendrocytes and neurons in schizophrenia. Moreover, CBD increased the voltage gated channel (CACNA1H) that is involved in cannabinoid retrograde signaling and glutamate and GABAergic neurotransmission. CACNA1H modulates Ca2+ levels and the synaptic vesicle cycle. To note, we also found effects of CBD on pathways and biological processes involved with schizophrenia pathobiology, such as glucose metabolism, axon guidance, and inflammation mediated by cytokine signaling. In summary, these proteomic findings may provide an integrated picture of the role of endocannabinoid signaling in oligodendrocyte cells and possible implications for schizophrenia’s pathobiology.

